# The impact mechanism of artificial intelligence dependence on college students’ innovation capability: an empirical study from China

**DOI:** 10.3389/fpsyg.2025.1732837

**Published:** 2025-12-12

**Authors:** Zhixin Yang, Hao Deng, Nan Jiang

**Affiliations:** School of Management, Guangxi Minzu University, Nanning, China

**Keywords:** artificial intelligence dependence, innovation capability, cognitive inertia, academic utilitarian atmosphere, employment pressure

## Abstract

**Introduction:**

The rapid adoption of artificial intelligence (AI) in higher education has increased college students’ reliance on AI tools. While AI enhances learning efficiency, it may also undermine key cognitive processes required for innovation.

**Methods:**

Using survey data from 1,032 students, this study employed partial least squares structural equation modeling (PLS-SEM) and fuzzy-set qualitative comparative analysis (fsQCA) to examine how AI dependence, cognitive inertia, employment pressure, and academic utilitarian atmosphere shape students’ innovation capability.

**Results:**

AI dependence significantly increases cognitive inertia, with cognitive dependence (*β* = 0.570, *p* < 0.001) exerting a stronger effect than tool dependence (*β* = 0.161, *p* < 0.001). Cognitive inertia reduces innovation capability (*β* = −0.111, *p* < 0.001) and serves as a key mediator linking AI dependence to innovation. Employment pressure strengthens the positive effect of AI dependence on cognitive inertia (*β* = 0.045, *p* < 0.05). A stronger academic utilitarian atmosphere further amplifies the negative impact of cognitive inertia on innovation capability (*β* = 0.052, *p* < 0.05). The fsQCA results reveal multiple pathways to high innovation capability, with low cognitive inertia emerging as a core condition across all effective configurations.

**Discussion:**

This study clarifies the cognitive mechanisms and contextual conditions through which AI dependence affects innovation. The findings extend research in educational technology and innovation psychology and offer practical guidance for universities to optimize learning environments, promote rational AI use, ease employment pressure, and improve academic culture.

## 1 Introduction

Innovation is the primary driving force behind technological progress. As the main contributors to future scientific and technological advancement, college students’ innovation capability not only serves as a core impetus for achieving breakthroughs in key technological fields and promoting high-quality economic development, but also represents an important indicator of higher education quality and the sustainability of innovation ecosystems. However, constrained by rigid traditional teaching models and excessive cognitive load, college students have long faced bottlenecks in innovative thinking and critical analysis, making it difficult to break through cognitive inertia and overcome limitations in originality. Against this backdrop, the rise of AI—particularly generative AI such as ChatGPT—offers new possibilities for addressing these challenges.

AI technology has brought significant changes to fields such as writing, research, and programming. Students’ use of AI is evolving from shallow functions like information retrieval and writing assistance to cognitive and decision-making processes, such as generating research outlines and assisting with data analysis (Himeur et al., 2023; Olsson and Engelbrektsson, 2022). This shift from instrumental use of AI to dependence, on the one hand, significantly improves learning and research efficiency (Qin et al., 2024); on the other hand, it may weaken students’ independent thinking and creativity (Zhai et al., 2024). In recent years, college students’ dependence on AI has been rising, yet there has been no significant improvement in academic innovation outcomes (Wang et al., 2025). This discrepancy highlights the tension between efficiency gains and depth of thinking, suggesting that AI may undermine students’ critical thinking and creativity (AlAli and Wardat, 2024).

Cognitive inertia emerges as a key clue to explain this contradiction. Cognitive inertia refers to individuals’ systematic avoidance of in-depth processing when facing complex or open-ended tasks, tending to rely on heuristics or ready-made answers (May, 2024). Existing studies have found that cognitive inertia is significantly negatively correlated with critical thinking, divergent thinking, and creative output, leading to lower scores in originality and practicality (Zhang et al., 2023). Once students become accustomed to relying on immediate feedback generated by AI technology, they are more likely to form an effort-saving learning model, weakening their exploratory investment and creative thinking (George, 2023). Therefore, clarifying whether AI affects innovation capability through cognitive inertia has become an urgent question for academia and educational practice to address.

Academic research has explored the relationship between AI and learning. Some studies focus on AI adoption and use, examining factors such as perceived usefulness, ease of use, and learning efficiency (Alshammari and Babu, 2025); others investigate AI’s impact on learning outcomes (Wu and Yu, 2024). However, conclusions in these two areas are conflicting: some studies suggest that AI improves efficiency and reduces burdens (Tong et al., 2023), while others argue it may weaken in-depth thinking and creativity (Wirawan and Saputri, 2024). Nevertheless, existing research has limitations: first, most studies remain at the level of AI use, lacking exploration of deeper psychological-behavioral phenomena related to AI dependence; second, academia primarily relies on short-term experiments or classroom interventions, with insufficient examination of long-term psychological mechanisms; third, the consequences of AI use are not unilinearly linear and may be significantly moderated by external contexts.

In the external environment, employment pressure and academic utilitarian atmosphere are two particularly critical factors. On one hand, with the growing risk of AI substitution and escalating job competition, college students generally feel resource threats and ability anxiety. Under high employment pressure, students are more inclined to adopt strategies to complete tasks quickly or with minimally acceptable quality, and AI, due to its efficiency and low cost, becomes a tool they frequently depend on (Esomonu, 2025). This pressure-driven dependence not only increases the baseline probability of AI use but also accelerates the formation of cognitive inertia through a preference for saving effort. On the other hand, the utilitarian orientation in university evaluation systems, which emphasizes the quantity of papers or competition results, drives students to use AI in a template-based manner and produce outputs quickly, thereby weakening originality and in-depth thinking (Duhaylungsod and Chavez, 2023).

Against this backdrop, this study focuses on three core research questions: (1) whether AI dependence affects cognitive inertia, (2) whether cognitive inertia mediates the effect of AI dependence on innovation capability, and (3) whether employment pressure and academic utilitarian atmosphere moderate this mechanism. The research contributions are mainly reflected in four aspects: first, it proposes a dual-dimensional framework distinguishing between tool dependence and cognitive dependence, breaking through the previous general treatment of AI use; second, it reveals that AI dependence indirectly inhibits innovation capability by strengthening cognitive inertia, indicating that technological convenience may reduce creative thinking; third, it analyzes the moderating roles of employment pressure and academic utilitarian atmosphere, clarifying the external environmental impact mechanism; fourth, it adopts a combined method of PLS-SEM and fsQCA, which not only verifies the robustness of the hypothetical paths but also identifies multiple conditional combinations for achieving high innovation capability, deepening the understanding of the formation mechanism of innovation capability from the perspective of multiple causes and multiple effects.

The structure of the article is as follows: Section 2 reviews the literature and proposes research hypotheses; Section 3 introduces the research methods; Section 4 presents the results; Section 5 discusses the findings; Section 6 puts forward implications and limitations; Section 7 concludes.

## 2 Literature review

### 2.1 Theoretical foundations

The popularization of AI is profoundly transforming the learning methods and cognitive structures of college students. Based on Social Cognitive Theory, there is a dynamic interactive relationship between individuals’ behaviors, cognitive states, and the environment (Riley et al., 2016). Within this framework, the use of AI tools not only changes how students acquire knowledge but also reshapes their thinking processes. When AI use evolves from an auxiliary tool to a cognitive substitution mechanism, students may form a dependent learning model, weakening active thinking and independent exploration, thereby exacerbating cognitive inertia. This shift from auxiliary to substitutive use reveals the potential tension between technology use and cognitive processes.

Technology Alienation Theory further deepens the understanding of cognitive dependence. According to existing definitions, when technology transforms from a purely empowering tool to a substitute thinking subject, individuals’ autonomy and subjective awareness may be weakened, leading to psychological dependence on technology (Lee and Lee, 2020). In contexts where AI is extensively involved in learning, students may tend to outsource thinking tasks to technical systems, undermining their own creative processing capabilities and critical reflection tendencies (Obaje, 2025). As this dependence gradually solidifies, cognitive inertia—defined as a state where individuals tend to rely on ready-made information and reduce active thinking—emerges as a typical psychological manifestation of technology alienation (Kuzmanov, 2025).

Conservation of Resources Theory posits that individuals tend to acquire, maintain, and protect limited resources, including psychological resources such as time, energy, and cognitive ability (Chen et al., 2018). When facing cognitive load or external pressure, individuals typically adopt resource-conserving strategies to minimize psychological effort (Guo et al., 2025). From this perspective, the convenience of AI enables students to more easily rely on external systems to complete complex tasks, reducing their own cognitive investment. However, excessive dependence may trigger cognitive inertia and further inhibit innovation capability. Therefore, there is a potential positive reinforcement relationship between AI dependence and cognitive inertia, and the inhibitory effect of cognitive inertia on innovation capability forms a key psychological mechanism chain.

External situational factors also play an important role in this mechanism. Situational Strength Theory suggests that when rules are clear and rewards and punishments are distinct in the environment, the consequences of individuals’ behaviors are more likely to be amplified (Stone and Jawahar, 2021). This means that the environment exerts a stronger constraining or facilitating effect on individuals’ decisions and behaviors. In an academically utilitarian atmosphere, students are more likely to focus on short-term outcomes and external evaluations, thereby exacerbating the adverse impact of cognitive inertia on innovation. Conversely, a supportive learning environment may buffer this negative effect by providing psychological and resource guarantees. As another typical strong situational variable, employment pressure further amplifies students’ resource-conserving tendencies, making it easier for AI dependence to transform into inertial thinking (Liu et al., 2025). Thus, the relationships between AI dependence, cognitive inertia, and innovation capability not only reflect the interactive process between technology and cognition but are also jointly shaped by academic and social environments.

In summary, Social Cognitive Theory reveals the behavioral and cognitive foundations of AI dependence; Technology Alienation Theory explains the evolutionary process from tool use to psychological dependence; Conservation of Resources Theory clarifies the internal motivations for inertia formation; and Situational Strength Theory uncovers the amplifying and moderating effects of the external environment on this mechanism. Together, these theories constitute the analytical framework of this study, providing a solid theoretical foundation for the subsequent proposal of hypotheses and model construction.

### 2.2 AI dependence and cognitive inertia

With the popularization of generative AI in higher education, students are increasingly relying on intelligent tools for learning tasks such as writing, programming, data processing, and information retrieval to reduce cognitive load and improve learning efficiency. However, unlike purely instrumental use, AI dependence is a stable psychological and behavioral tendency, characterized by actively turning to external intelligent systems for in-depth processing tasks and using AI as a continuous source of cognitive and functional support (Aghaziarati and Rahimi, 2025). In other words, AI dependence emphasizes psychological reliance rather than mere usage frequency, tool utility, or short-term cognitive offloading.

This study divides AI dependence into two dimensions: first, tool dependence, which reflects the degree to which students rely on functional assistance (such as operation, retrieval, and generation) provided by AI tools when completing learning tasks, belonging to task-level functional support (Shao et al., 2025). For example, students who frequently rely on AI to generate first drafts or integrate references when writing papers exhibit tool dependence (Plathottam et al., 2023). Second, cognitive dependence, which refers to students’ tendency to rely on AI to replace independent thinking in high-level cognitive activities such as understanding, reasoning, conception, and judgment, belonging to cognitive support at the psychological processing level (da Silva, 2024). For example, students who rely on AI to provide logical reasoning or decision-making suggestions when solving complex problems—rather than conducting independent analysis and judgment—exhibit cognitive dependence (Zhai et al., 2024).

AI dependence may gradually solidify into cognitive inertia. Cognitive inertia refers to an individual’s tendency to maintain existing cognitive structures, judgment patterns, and behavioral preferences when confronted with new information, technologies, or situations, showing reluctance to adjust or change. Its essence lies in a stable form of cognitive path dependence, whereby individuals rely on familiar experiences to interpret external stimuli, leading to rigidity in evaluation, decision-making, and technology adoption (Szmyd and Mitera, 2024). According to the cognitive miser theory, when individuals can effortlessly accomplish tasks using low-cost and highly efficient AI tools, their instinct to minimize cognitive investment is activated, leading to systematic avoidance of deep processing—an essential behavioral manifestation of cognitive inertia (Szmyd and Mitera, 2024). Social Cognitive Theory further explains that this reliance becomes increasingly reinforced and normalized through widespread peer adoption and environmental cues, transforming from a habitual tool-use pattern into a stable tendency toward cognitive inertia (Constantino et al., 2022). This transition from dependence to inertia is particularly evident in specific learning mechanisms. At the metacognitive level, cognitive reliance weakens learners’ abilities in autonomous planning and monitoring (Babayev, 2025), diminishing their awareness and regulation of their own thinking. At the level of cognitive effort, excessive reliance directly leads to insufficient deep processing of complex problems (Elzerman, 2025). The weakening of these key learning mechanisms jointly creates fertile conditions for the development of cognitive inertia (Frenkenberg and Hochman, 2025).

A growing body of empirical research has observed the outcomes of this transition pathway. Studies have found that frequent AI intervention is accompanied by declines in students’ critical reasoning and active processing abilities, giving rise to a “generate first, think later” behavioral pattern (Szmyd and Mitera, 2024). This pattern represents a typical form of cognitive inertia, in which individuals facing complex tasks no longer initiate independent thinking but instead habitually seek and rely on ready-made answers (May, 2024).

In summary, while the convenience of AI reduces surface-level cognitive load, it may simultaneously weaken learners’ deeper cognitive capacities, fostering a reliance-based cognitive inertia that ultimately alters the cognitive processes and behavioral patterns essential for innovation. Based on this, we propose the following hypothesis:

*Hypothesis 1 (H1)*: AI dependence has a significant positive impact on the cognitive inertia of college students.

*H1a*: Tool dependence has a significant positive impact on the cognitive inertia of college students.

*H1b*: Cognitive dependence has a significant positive impact on the cognitive inertia of college students.

### 2.3 Cognitive inertia and innovation capability

Innovation capability refers to an individual’s ability to generate novel and effective solutions through critical reasoning, divergent thinking, and cross-domain integration when facing complex or open-ended problems (Gao et al., 2025). Innovative activities are usually accompanied by high levels of cognitive load and uncertainty, requiring sustained attention investment and in-depth processing. However, some learners tend to avoid mental effort when facing complex cognitive tasks, relying on familiar paths or external cues, a tendency known as cognitive inertia (May, 2024).

Cognitive inertia is essentially a cognitive resource conservation strategy, characterized by surface-level processing, template-based expression, and insufficient metacognitive monitoring (Wu, 2025). Existing studies have shown that cognitive inertia is significantly negatively correlated with critical thinking, problem restructuring, and divergent thinking (Wu et al., 2020), and is manifested as low scores in originality, fluency, and practicality in creativity assessments (Frith et al., 2021). The fundamental mechanism is that cognitive inertia weakens exploratory search and multi-solution evaluation, inhibits curiosity and cognitive needs, leading individuals to adopt passive response strategies when facing innovative tasks, thereby limiting the breadth and depth of idea generation.

In the process of learning and creation, the formation of innovation capability relies on continuous cognitive investment and reflective processing, while cognitive inertia weakens this process by reducing thinking activity and exploration motivation. If students rely on simplified thinking modes or inertial responses for a long time, their thinking flexibility and creative processing capabilities will gradually decline (Fuchs et al., 2023). It can be seen that cognitive inertia is not only a negative psychological state but also a core internal factor restricting the development of college students’ innovation capability.

*Hypothesis 2 (H2)*: Cognitive inertia has a significant negative impact on the innovation capability of college students.

### 2.4 Mediating role of cognitive inertia

Cognitive inertia plays a key mediating role between AI dependence and innovation capability, and its mechanism is not a simple linear one but reflects a differential pattern of moderate impact and excessive inhibition. Moderate use of AI can reduce cognitive load and improve learning efficiency, thereby releasing resources for higher-order thinking and creative processing (Chandrasekera et al., 2025). However, when the frequency of use is too high or the degree of dependence is too deep, students are more likely to rely on externally generated results rather than active thinking, leading to a decline in in-depth processing and original performance (Zhai et al., 2024).

Cognitive inertia provides a psychological mechanism explanation for this phenomenon. Studies have shown that cognitive inertia is significantly negatively correlated with critical thinking and divergent thinking (Wu et al., 2020), and is closely related to the decline in the number of ideas, originality, and practicality (Zhou et al., 2022). In the AI context, learning models that rely on immediate feedback are more likely to form a tendency to save effort, reducing opportunities for independent analysis and creative exploration (Grassini, 2023). Social Cognitive Theory points out that an individual’s behavior and cognition are reinforced by the external environment, and high-frequency AI use may be internalized into an inertial thinking mode (Kuzmanov, 2025). Conservation of Resources Theory further explains that when individuals perceive cognitive load or limited psychological resources, they tend to rely on external tools to save resources (Hobfoll and Freedy, 2017). At the same time, Technology Alienation Theory holds that when AI transforms from a cognitive enhancement tool to a thinking substitute, students’ subjectivity is weakened, and a dependent mode of generating first and then thinking is gradually formed (George, 2023).

Cognitive inertia is both a psychological outcome of AI dependence and a key transmission mechanism through which it impairs innovation capability. AI dependence indirectly affects students’ creative performance by inducing inert thinking, forming an important path affecting innovation capability. Therefore, cognitive inertia is not only a direct result of AI but also a key mediating mechanism through which it weakens innovation capability.

*Hypothesis 3 (H3)*: AI dependence indirectly affects the innovation capability of college students through cognitive inertia.

### 2.5 Moderating role of employment pressure

Employment pressure is an important situational variable affecting college students’ learning strategies and innovative behaviors. Currently, college students generally face two types of pressure sources: one is career substitution anxiety caused by the rapid development of AI, and the other is psychological burden brought by the increasingly fierce competition in the real job market. This dual pressure makes students perceive incompetence and resource scarcity at the cognitive and emotional levels, forming a obvious sense of resource threat.

According to Conservation of Resources Theory, when individuals perceive external threats or resource scarcity, they will prioritize resource-saving strategies to maximize benefits and minimize consumption (Hobfoll and Freedy, 2017). In learning scenarios, this strategy is often manifested as relying on external technical tools to complete tasks to reduce time and cognitive investment. Due to their characteristics of immediate feedback and efficient generation, AI tools have become the preferred resource for students under high-pressure conditions. However, long-term dependence on AI assistance may weaken learners’ independent processing and in-depth thinking capabilities, thereby consolidating cognitive inertia (Lin et al., 2025).

Employment pressure is not only an external situational background in the learning process but also a key moderating factor that amplifies the relationship between AI dependence and cognitive inertia. When students are under high employment pressure, they are more inclined to seek efficient solutions through AI tools to alleviate psychological and cognitive burdens (Li and Jan, 2023), and this behavior further strengthens the psychological mechanism of replacing cognitive investment with efficiency. In contrast, in a low-pressure environment, students may be more willing to maintain independent thinking and exploration behaviors, reducing the tendency to use AI tools inertly (Zhang, 2025). This study argues that employment pressure significantly enhances the positive effect of AI dependence on cognitive inertia by strengthening the motivation to conserve resources. This mechanism is reflected in two specific paths: on the one hand, high employment pressure will amplify the impact of AI cognitive dependence (i.e., thinking dependence on AI judgments and generated results) on cognitive inertia; on the other hand, it will also strengthen the inducing effect of AI tool dependence (i.e., use dependence on AI functional operations and technical convenience) on inert behaviors.

*Hypothesis 4 (H4)*: Employment pressure plays a moderating role between AI dependence and cognitive inertia.

*H4a*: Employment pressure positively moderates the relationship between AI cognitive dependence and cognitive inertia.

*H4b*: Employment pressure positively moderates the relationship between AI tool dependence and cognitive inertia.

### 2.6 Moderating role of academic utilitarian atmosphere

The academic utilitarian atmosphere is an important external situational factor in the college educational environment, characterized by overemphasizing quantifiable achievement indicators such as the number of papers, competition results, and ranking performance. This utilitarian-oriented evaluation system strengthens students’ pursuit of short-term goals and weakens their exploratory and original tendencies in the learning process (Mpolomoka, 2025). In this context, students tend to choose efficiency-oriented learning strategies to meet external evaluation standards rather than conducting in-depth processing and independent innovation.

According to Situational Strength Theory, in strong situations with clear rewards and punishments and clear norms, individual behavior is more likely to be driven by the external environment, and its consequences are significantly amplified (Dalal et al., 2015). The utilitarian academic atmosphere in colleges and universities is a typical example of such a strong situation. By strengthening the external reward and punishment mechanism, it makes students more dependent on template-based generation and immediate output in learning and scientific research, gradually weakening their ability of thinking reflection and knowledge integration.

Existing empirical studies have found that in colleges or disciplines with a stronger utilitarian atmosphere, the novelty, originality, and creative output of students’ research questions are significantly reduced (Fülöp et al., 2023). This indicates that the academic utilitarian atmosphere not only affects learning motivation but also resonates with cognitive inertia, amplifying the inhibitory effect of inert thinking on innovative performance. In other words, the utilitarian orientation drives short-termism through evaluation pressure, further prompting students to rely on surface-level output and template-based thinking, thereby strengthening the adverse impact of cognitive inertia on innovation and exacerbating its negative effects downstream of the AI dependence path.

*Hypothesis 5 (H5)*: The academic utilitarian atmosphere plays a moderating role in the relationship between cognitive inertia and the innovation capability of college students.

**Figure 1:**
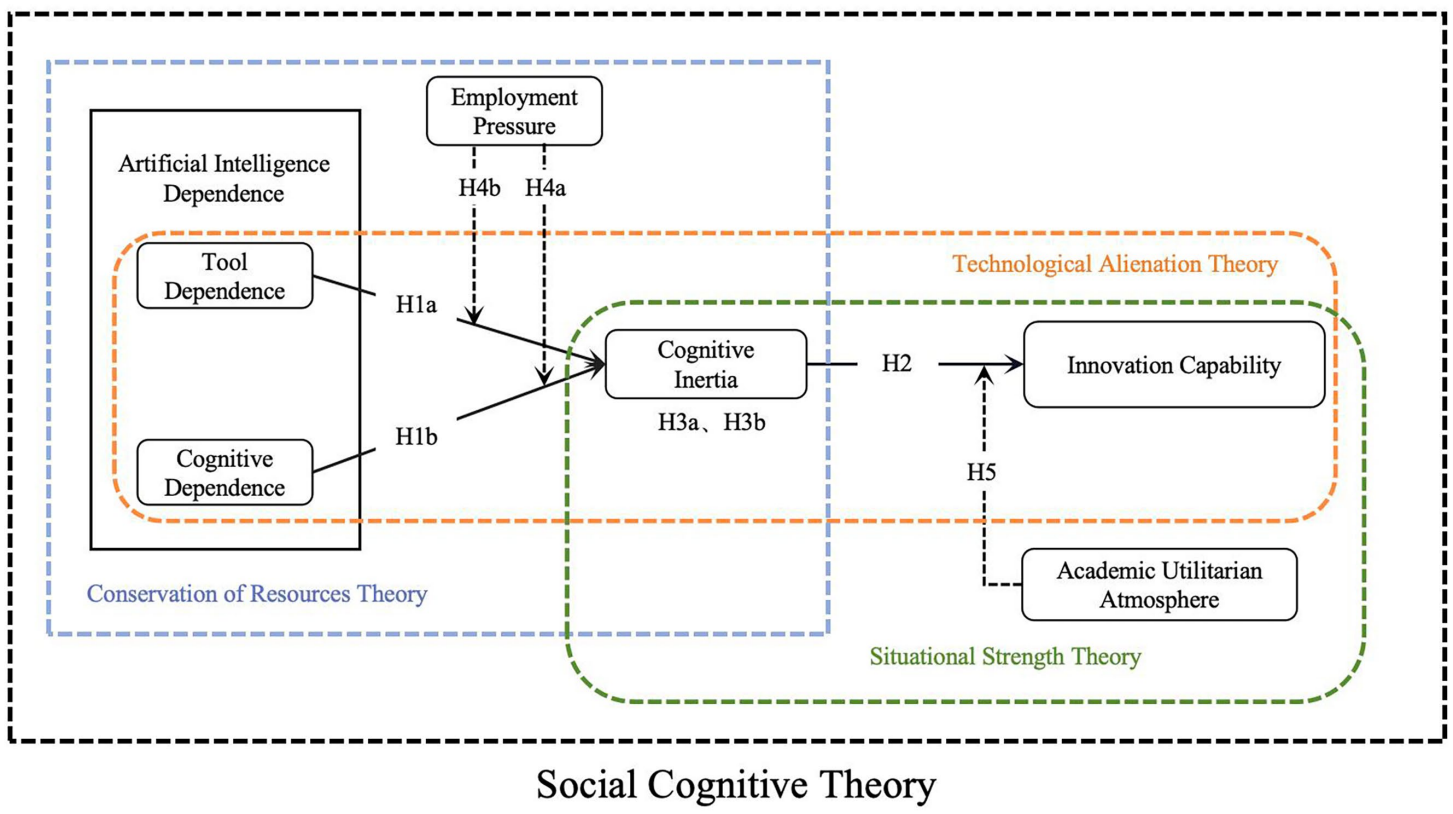
Theoretical model and research hypotheses.

Figure 1 presents the theoretical logic and hypothesized pathways of this study, forming an integrated model of college students’ innovation capability. The model conceptualizes AI dependence along two dimensions and positions it as an antecedent of cognitive inertia. Through the core mechanism linking “AI dependence → cognitive inertia → innovation capability,” the model illustrates how reliance on AI shapes students’ cognitive processes and ultimately their innovative performance. To further contextualize this mechanism, two external contextual factors—employment pressure and academic utilitarian climate—are incorporated to examine their moderating roles.

Beyond depicting structural pathways, this framework provides a more comprehensive conceptual integration. Grounded in the triadic interaction of person–behavior–environment in Social Cognitive Theory, the model synthesizes key insights from Conservation of Resources Theory, Technological Alienation Theory, and Situational Strength Theory. This integration clarifies how cognitive inertia emerges as a resource-conserving yet potentially maladaptive cognitive strategy, how AI dependence may induce forms of cognitive offloading and technocratic thinking, and how contextual pressures strengthen or weaken these processes.

The theoretical contribution of this framework lies in three aspects. First, it advances the conceptualization of cognitive inertia by situating it within technology-assisted learning contexts, enriching existing decision-making–oriented definitions. Second, it links AI dependence to cognitive inertia, providing a novel mechanism explaining how emerging technologies may inadvertently hinder innovation. Third, it incorporates contextual forces to illuminate when and why these mechanisms intensify, offering a more nuanced and ecologically valid understanding of students’ innovation capability.

## 3 Materials and methods

### 3.1 Research design

This study adopts a quantitative research design to systematically examine the mechanism through which AI dependence, cognitive inertia, employment pressure, and academic utilitarian atmosphere affect college students’ innovation capability. Given that the research topic involves interactive effects of psychological tendencies, behavioral patterns, and environmental perceptions, the quantitative questionnaire method can quantify individual differences in a standardized manner, ensuring data reliability, comparability, and statistical inference validity (Mellinger and Hanson, 2020).

The questionnaire consists of two main parts: The first part includes respondents’ basic demographic information, such as gender, grade, academic discipline, academic performance, practical experience, and participation in student work. These variables are incorporated as control variables in subsequent statistical analyses to reduce potential confounding effects and enhance the model’s explanatory power. The second part measures core research variables, including: (1) AI dependence, covering two dimensions of tool dependence and cognitive dependence; (2) cognitive inertia, measuring individuals’ tendency to avoid in-depth processing and rely on ready-made answers when facing complex tasks; (3) innovation capability, assessing students’ abilities in divergent thinking, problem-solving, and originality; (4) employment pressure, measuring students’ perceptions of career competition and future employment uncertainty; (5) academic utilitarian atmosphere, reflecting students’ cognition of the environment in colleges that overemphasizes rankings and achievement orientation. All items for variable measurement are evaluated using a 5-point Likert scale.

In terms of research methods, this study adopts a complementary design by combining PLS-SEM and fsQCA. PLS-SEM is used to test theoretical hypotheses and estimate direct effects, mediating effects, and moderating effects, helping to reveal the linear influence mechanisms between variables; fsQCA, based on set theory, can identify combination paths of multiple antecedent conditions and uncover the multi-causal synergistic logic underlying the formation of innovation capability. The innovation of this method integration lies in: PLS-SEM focuses on average effects and is suitable for verifying theoretical deductions; fsQCA emphasizes multi-path causal equivalence and is applicable to the conditional combination analysis of complex social behaviors. Their combination not only makes up for the limitations of a single statistical model but also enables this study to deepen the understanding of how AI use, cognitive traits, and situational factors jointly affect innovation capability from both linear causal mechanisms and complex configuration mechanisms, thereby enhancing the comprehensiveness of theoretical explanations and the robustness of results.

### 3.2 Data collection

This study collects data through a questionnaire survey. Compared with interview or experimental research, questionnaire surveys can systematically obtain individuals’ psychological and behavioral characteristics within a large sample range, with advantages such as simple operation, low cost, and strong statistical comparability (Lund, 2023). Meanwhile, this method helps ensure data anonymity, reduce social desirability bias, and improve measurement reliability and validity through standardized items, making it suitable for exploring college students’ learning behaviors and cognitive characteristics.

In terms of sampling, this study adopted a stratified convenience sampling method. First, taking university hierarchy and regional distribution as the first-level stratification criteria, comprehensive universities, science and engineering universities, and normal universities in eastern and central China were selected to obtain responses from students of different types of universities; second, academic disciplines were used as the second-level stratification criteria, covering three categories of majors: science and engineering, management, and humanities, to increase the diversity of the data in terms of disciplinary composition. The above strategies strive to improve the diversity of the sample structure on the basis of convenience sampling, but may still be affected by biases caused by self-selected samples and regional limitations, for example, failing to cover students from universities in western China and high-level institutions.

The questionnaires were distributed online through the Wenjuanxing platform, and a total of 1,100 questionnaires were collected. After excluding 68 invalid questionnaires with excessively short filling time (less than 120 s) and missing items, 1,032 valid samples were finally obtained, with an effective rate of 93.82%. The sample showed a certain degree of distribution balance in terms of gender, grade, and disciplinary category. However, due to the use of convenience sampling, the results of this study are more suitable for understanding the correlation patterns between AI dependence, cognitive inertia, and innovation capability among college students within this sample, and should not be directly generalized to all Chinese college student groups. Future research can further enhance the generalizability of the research conclusions through probability sampling or large-sample surveys across multiple regions.

### 3.3 Analytical methods

In this study, PLS-SEM is used to test the relationships among AI dependence, cognitive inertia, and innovation capability, and further examine the moderating roles of employment pressure and academic utilitarian atmosphere. PLS-SEM has significant advantages in handling complex theoretical models (Assaker and O’Connor, 2023), which helps to systematically present the effect chain of AI dependence influencing innovation capability through cognitive inertia and to examine the roles of external situational variables. Second, it has relatively loose requirements for sample size and distribution, does not rely on the assumption of normal distribution, and is suitable for the data analysis of this study based on 1,032 valid questionnaires. Third, it can not only test the significance of path coefficients but also evaluate the predictive validity and explanatory power of the model, providing robust statistical support for the research conclusions.

However, PLS-SEM mainly relies on linear models and tests of average effects, making it difficult to capture the phenomena of multiple causes leading to one result and the same cause leading to different results, which are common in educational practice. To make up for this limitation, this study further introduces fsQCA. Different from variable-centered methods, fsQCA is based on set theory logic and can identify how different conditional combinations jointly shape students’ learning and innovation outcomes (Rasoolimanesh et al., 2023).

This study adopts a complementary analytical strategy of PLS-SEM and fsQCA to organically combine path testing and conditional configuration analysis. PLS-SEM is suitable for verifying path relationships in the theoretical model and their significance, while fsQCA can reveal multiple antecedent combinations under different contexts. Their combination not only improves the explanatory power and external validity of the study but also helps more comprehensively reveal the mechanism and boundary conditions of AI dependence on college students’ innovation capability. This multi-method integration paradigm provides a more in-depth methodological approach for research on educational technology and cognitive mechanisms. The detailed introductions of the two methods are shown in Appendix 1.

### 3.4 Demographics

**Figure 2:**
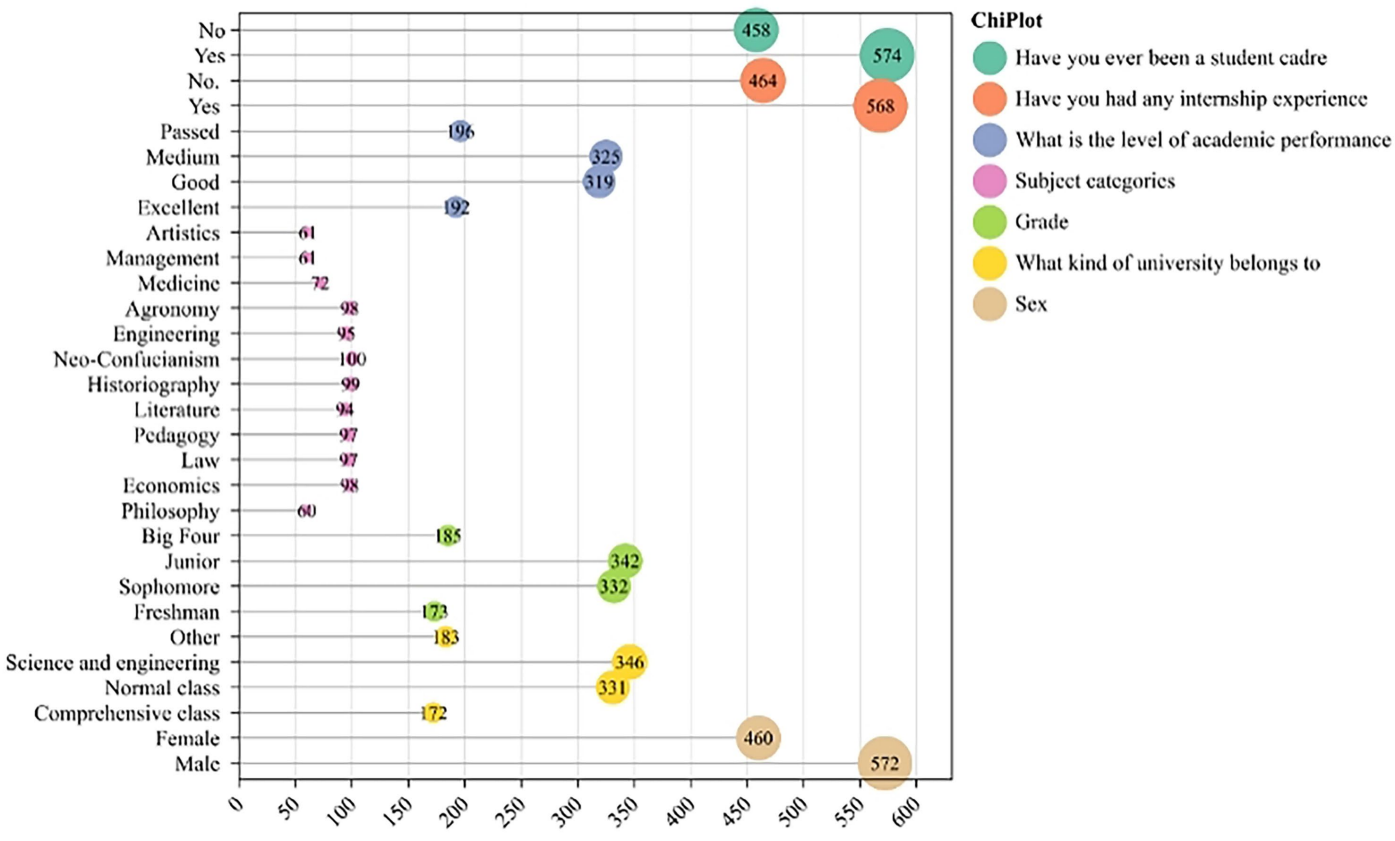
Distribution of basic sample information.

Figure 2 presents the basic characteristics of the sample in this study (N = 1,032), including gender, grade, and major category. The overall distribution is relatively balanced, covering different learning stages and major disciplinary types, indicating that the sample structure has good representativeness. Among the participants, 55.4% were male and 44.6% were female, with a slightly higher proportion of males. In terms of university types, 33.5% were from science and engineering universities, 32.1% from normal universities, 16.7% from comprehensive universities, and 17.7% from other types, covering mainstream institutional categories. Regarding academic grades, sophomores accounted for 32.1% and juniors for 33.0%, with approximately 17% in each of the lower and upper grade levels, aligning with the critical period of professional cognitive development. The sample covered 12 academic disciplines: natural sciences, history, economics, agriculture, law, and education each accounted for approximately 9.5%, while medicine, philosophy, management, and art each accounted for around 6%. In terms of academic performance, 31.5% were rated as average, 30.9% as good, 18.6% as excellent, and 19.0% as passing, showing a normal distribution. For practical experience, 55.0% had internship experience, 55.6% had served as student leaders, and over 50% had both types of experience.

## 4 Results

### 4.1 Descriptive statistics and correlation analysis

#### 4.1.1 Descriptive analysis and normality test

According to the descriptive analysis in Table 1, the core variables of this study exhibited distinct distribution characteristics among the 1,032 valid samples. In terms of central tendency, the mean values of all variables were higher than 3, with employment pressure being the highest (4.050) and college students’ innovation capability being the lowest (3.764). This indicates that students experience relatively prominent employment pressure, while their innovation capability is slightly insufficient. The mean values of AI tool dependence, AI cognitive dependence, cognitive inertia, academic utilitarian atmosphere, and employment pressure all ranged between 3.8 and 4.1, reflecting significant characteristics of students in these dimensions.

**Table 1:** Descriptive analysis of variables.

Variable	*N*	Minimum	Maximum	Mean	Standard deviation
AI tool dependence	1,032	1	5	4.038	0.657
AI cognitive dependence	1,032	1	5	3.969	0.853
Cognitive inertia	1,032	1	5	3.968	0.889
Academic utilitarian atmosphere	1,032	1	5	3.860	0.853
Employment pressure	1,032	1.222	5	4.050	0.724
Innovation capability	1,032	1	5	3.764	0.806

#### 4.1.2 Correlation analysis

**Figure 3:**
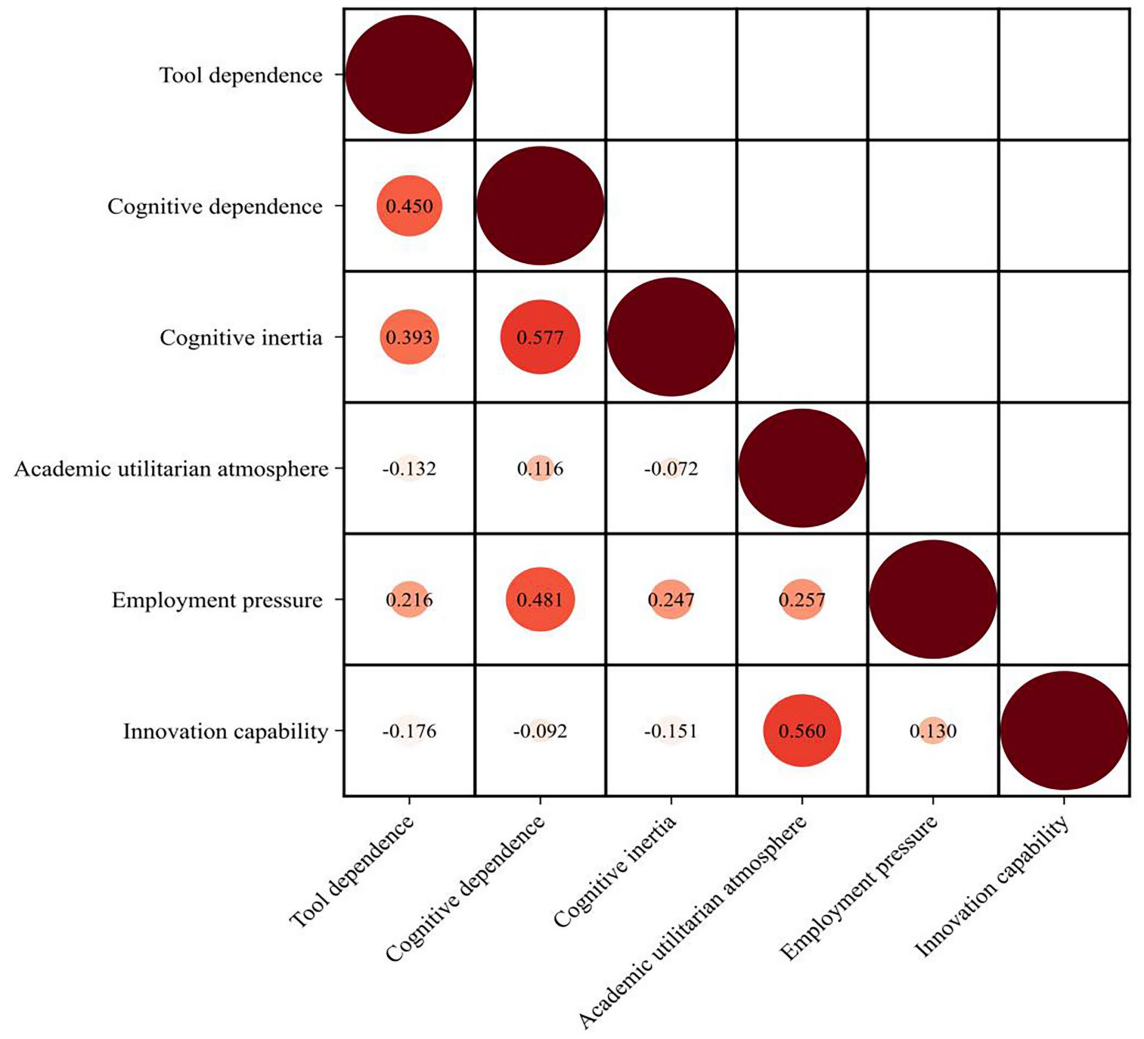
Correlation analysis.

As shown in Figure 3, AI tool dependence was significantly positively correlated with cognitive inertia (*r* = 0.393, *p* < 0.01), and cognitive dependence showed a stronger positive correlation with cognitive inertia (*r* = 0.577, *p* < 0.01). This indicates that students’ dependence both in tool use and cognitive aspects may promote the formation of cognitive inertia, with the impact of cognitive dependence being more prominent. Meanwhile, cognitive inertia was significantly negatively correlated with innovation capability (*r* = −0.151, *p* < 0.01), confirming that cognitive inertia may inhibit in-depth thinking and innovative exploration.

### 4.2 Measurement model test

According to the test results (Appendix 2), the mean values of all items in the scales of this study ranged from 3.66 to 4.16, with standard deviations between 0.925 and 1.213. Normality test results showed that the skewness and kurtosis of all items were within acceptable ranges, with little deviation from the normal distribution. The data distribution of the scale items was reasonable, allowing for further model test analysis. In addition, in the multicollinearity test (Appendix 3), the VIF values of all items ranged from 1.608 to 3.046, all far below the critical value of 5, indicating no serious multicollinearity issues.

Before conducting structural model analysis, reliability and validity tests of the measurement model were required, including Cronbach’s *α*, composite reliability, average variance extracted (AVE), and factor loadings, all tested using the Bootstrapping method (cases = 749, samples = 5,000). As shown in Table 2, the Cronbach’s α coefficients of each latent variable ranged from 0.877 to 0.959, all significantly higher than the critical value of 0.7, indicating good internal consistency of the scales. The composite reliability ranged from 0.897 to 0.962, all exceeding the ideal standard of 0.8, demonstrating stability and reliability of the measurement results. The AVE values of each latent variable ranged from 0.505 to 0.609, all meeting the minimum requirement of 0.5, indicating that the items could effectively explain the core connotation of the latent variables, with acceptable convergent validity. In addition, all items in the scales had factor loadings on their corresponding latent variables ranging from 0.601 to 0.830, all exceeding the threshold of 0.6, and no cross-loadings were observed (Appendix 3), indicating good measurement validity of the scales. The measurement model of this study meets academic standards in terms of reliability and validity, and can support subsequent structural model analysis.

**Table 2:** Cronbach’s α, composite reliability, and convergent validity.

Variable	Cronbach’s alpha	Composite reliability (rho_c)	Average variance extracted (AVE)
Innovation capability	0.959	0.962	0.505
Academic utilitarian atmosphere	0.909	0.926	0.580
Employment pressure	0.882	0.904	0.513
Tool dependence	0.877	0.897	0.522
Cognitive dependence	0.912	0.928	0.587
Cognitive inertia	0.921	0.933	0.609

As shown in Table 3, the measurement model of this study had good discriminant validity, ensuring the independence between different variables. According to the Fornell–Larcker criterion (Yusoff et al., 2020), the square root of AVE for each latent variable was greater than its correlation coefficients with other variables. Each construct in the model had clear boundaries, and the measurement dimensions were reasonably differentiated.

**Table 3:** Discriminant validity.

Variable	Innovation capability	Academic utilitarian atmosphere	Employment pressure	Tool dependence	Cognitive dependence	Cognitive inertia
Innovation capability	0.710					
Academic utilitarian atmosphere	0.587	0.762				
Employment pressure	0.151	0.258	0.716			
Tool dependence	−0.160	−0.110	0.239	0.649		
Cognitive dependence	−0.062	0.116	0.485	0.473	0.766	
Cognitive inertia	−0.137	−0.040	0.279	0.414	0.608	0.780

### 4.3 Structural model test

The evaluation of the structural model not only involves testing the validity of the structural model but also verifying whether the relationships defined in the theoretical construction stage hold. When evaluating the model, it is first necessary to assess the predictive effect of the measurement equation; if the predictive effect of the measurement equation is poor, the evaluation of the structural equation will lose its significance. In Smart-PLS 4.0, common evaluation indicators include *R*^2^, SRMR, and NFI. *R*^2^ measures the explanatory power of independent variables on endogenous latent variables, with larger values indicating better model validity; generally, 0.02, 0.13, and 0.26 represent low, medium, and high levels of explanatory power, respectively (Samartha, 2020). SRMR reflects the residual fit of the model, with values less than 0.1 indicating a good fit; NFI is better when closer to 1, and generally, values greater than 0.7 are acceptable. As shown in Table 4, the *R*^2^ values of innovation capability and cognitive inertia are both higher than 0.26, indicating strong explanatory power; meanwhile, SRMR < 0.1 and NFI > 0.7, suggesting that the structural model of this study has good validity.

**Table 4:** Fit indices.

Variable	*R*-square	*R*-square adjusted	SRMR	NFI
Innovation capability	0.362	0.360	0.068	0.752
Cognitive inertia	0.409	0.407		

**Figure 4:**
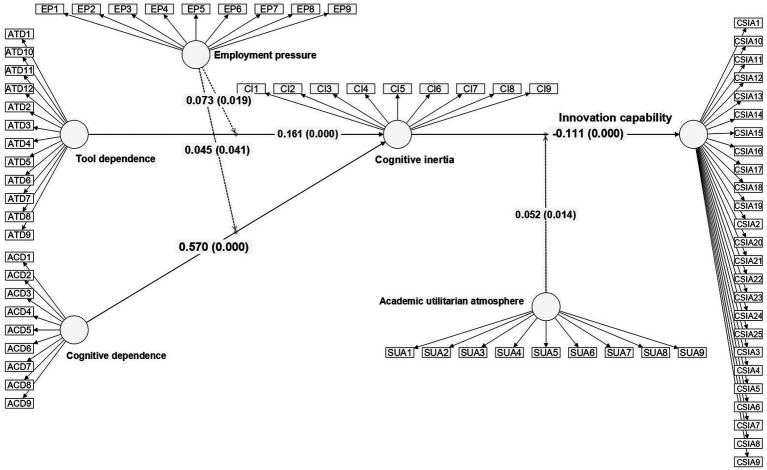
Results of structural model test.

Figure 4 further presents the path test results of the structural model, including the standardized coefficients and significance levels of the main effects and moderating effects. The results show that both tool dependence and cognitive dependence have a significant positive impact on cognitive inertia, and cognitive inertia exerts a significant negative effect on innovation capability; employment pressure significantly enhances the impact of dependence on cognitive inertia, while the academic utilitarian atmosphere strengthens the inhibitory effect of cognitive inertia on innovation capability. Overall, Figure 4 illustrates the statistical support for the hypothesized paths in this study, providing visual evidence for the validity of the theoretical model.

As shown in the hypothesis test results of the structural equation model in Table 5, all research hypotheses are supported. In terms of direct effects: tool dependence has a significant positive predictive effect on cognitive inertia (*β* = 0.161, *p* < 0.001), thus Hypothesis H1a is supported; cognitive dependence has a stronger positive driving effect on cognitive inertia (*β* = 0.570, *p* < 0.001), verifying Hypothesis H1b; cognitive inertia has a significant negative impact on innovation capability (*β* = −0.111, *p* < 0.001), indicating that the higher the level of cognitive inertia, the weaker the individual’s innovation capability, thus Hypothesis H2 is validated.

**Table 5:** Test results.

No.	Path	*β*	SD	*T*	*P*	Decision
H1a	Tool dependence → Cognitive inertia	0.161	0.028	5.812	0	Supported
H1b	Cognitive dependence → Cognitive inertia	0.570	0.040	14.317	0	Supported
H2	Cognitive inertia → Innovation capability	−0.111	0.025	4.451	0	Supported
H3a	Tool dependence → Cognitive inertia → Innovation capability	−0.018	0.005	3.757	0	Supported
H3b	Cognitive dependence → Cognitive inertia → Innovation capability	−0.063	0.015	4.151	0	Supported
H4a	Employment pressure × Cognitive dependence → Cognitive inertia	0.045	0.022	2.041	0.041	Supported
H4b	Employment pressure × Tool dependence → Cognitive inertia	0.073	0.031	2.352	0.019	Supported
H5	Academic utilitarian atmosphere × Cognitive inertia → Innovation capability	0.052	0.021	2.464	0.014	Supported

Regarding mediating effects: tool dependence can indirectly exert a negative impact on innovation capability through cognitive inertia (*β* = −0.018, *p* < 0.001), supporting Hypothesis H3a; the negative mediating effect of cognitive dependence on innovation capability through cognitive inertia is more obvious (*β* = −0.063, *p* < 0.001), thus Hypothesis H3b is supported, which suggests that the indirect inhibitory effect of cognitive dependence on inn.

At the level of moderating effects, employment pressure significantly and positively moderates the relationship between cognitive dependence and cognitive inertia (*β* = 0.045, *p* < 0.05). Specifically, the greater the employment pressure, the stronger the positive impact of cognitive dependence on cognitive inertia, thus Hypothesis H4a is supported. Meanwhile, employment pressure also significantly and positively moderates the relationship between tool dependence and cognitive inertia (*β* = 0.073, *p* < 0.05), verifying Hypothesis H4b; in addition, the academic utilitarian atmosphere significantly and positively moderates the relationship between cognitive inertia and innovation capability (*β* = 0.052, *p* < 0.05), indicating that in an environment with a stronger utilitarian atmosphere, the negative inhibitory effect of cognitive inertia on innovation capability will be further strengthened, thus Hypothesis H5 is validated.

### 4.4 Fuzzy-set qualitative comparative analysis

This study first used partial least squares structural equation modeling to test the relationships between core variables and moderating effects. Its advantages lie in the ability to simultaneously analyze direct, mediating, and moderating effects, having loose requirements for sample distribution, and enabling evaluation of model validity. However, PLS-SEM is based on variable-centered linear logic, making it difficult to address the causal complexity in the formation of innovation capability: it cannot capture configuration characteristics such as multiple causes leading to one result or the same cause leading to different results, nor can it reveal dynamic interaction effects among multiple variables, let alone answer practical questions about how to achieve high innovation through conditional combinations. Therefore, this study introduced fsQCA: based on configuration-centered set theory logic, it can identify multiple antecedent configurations for high innovation capability, reflect causal equivalence, and locate optimal conditional combinations through consistency and coverage indicators, which aligns with the needs of student heterogeneity and differentiated cultivation in colleges and universities.

#### 4.4.1 Necessity analysis

As shown in Table 6, this study calibrated six core variables (tool dependence, cognitive dependence, cognitive inertia, academic utilitarian atmosphere, employment pressure, and innovation capability) using a three-point scale in fsQCA. The calibration criteria for membership were: 95% for full membership, 50% for the crossover point, and 5% for non-full membership. These membership criteria were combined with the theoretical value range of the scales and the actual sample distribution, ensuring reasonable definitions of high and low levels. Overall, the calibration results are highly consistent with the scale characteristics and sample distribution, providing a robust set relationship basis for subsequent configuration analysis.

**Table 6:** Variable calibration.

Variable	Full membership	Crossover point	Non-full membership
Tool dependence	5.000	4.083	2.917
Cognitive dependence	4.889	4.222	1.739
Cognitive inertia	5.000	4.111	2.000
Academic utilitarian atmosphere	4.667	4.111	1.778
Employment pressure	4.889	4.222	2.000
College students’ innovation capability	4.680	4.000	2.000

In fsQCA, necessity analysis is a core preliminary step in configuration analysis. Its main purpose is to test whether a single antecedent condition is a necessary condition for achieving the target outcome—i.e., to determine if there exists a condition that, if absent, makes it absolutely impossible to achieve the target outcome regardless of other conditional combinations. This test directly determines the logical starting point and analytical direction of subsequent configuration analysis, with the judgment criterion that the consistency value must not exceed 0.9.

As shown in Table 7, this study verified the relationships between five core variables and college students’ innovation capability through fsQCA-based necessity analysis. The results showed that in the test for high innovation capability, the consistency values of all variables and their non-sets did not meet the 0.9 threshold for necessary conditions, with the highest being the academic utilitarian atmosphere (0.781). In the test for low innovation capability, the non-set consistency of cognitive dependence was the highest (0.748), while other variables also did not meet the standard. Coverage results were also at a moderate level (0.589–0.748), indicating that a single variable has a limited explanatory range for innovation capability. In summary, the study confirmed that no single variable constitutes a necessary condition for high or low innovation capability; college students’ innovation capability more relies on the synergistic effect of multiple variable combinations, providing a basis for subsequent configuration analysis.

**Table 7:** Necessity analysis.

Variable	High innovation capability		Low innovation capability	
	Consistency	Coverage	Consistency	Coverage
Tool dependence	0.672	0.673	0.729	0.670
~Tool dependence	0.671	0.730	0.645	0.643
Cognitive dependence	0.706	0.703	0.749	0.684
~Cognitive dependence	0.683	0.748	0.675	0.678
Cognitive inertia	0.725	0.697	0.759	0.669
~Cognitive inertia	0.656	0.748	0.656	0.686
Academic utilitarian atmosphere	0.781	0.745	0.673	0.589
~Academic utilitarian atmosphere	0.569	0.655	0.709	0.748
Employment pressure	0.745	0.723	0.731	0.651
~Employment pressure	0.640	0.722	0.689	0.713

#### 4.4.2 Configuration analysis

Table 8 systematically identifies the configurations for high and low innovation capability among college students based on fsQCA, providing important evidence for revealing the multi-causal synergistic mechanism underlying the formation of innovation capability. From the perspective of overall indicators, the consistency values of all configurations range from 0.861 to 0.923, significantly higher than the 0.8 robustness threshold in fsQCA methodology, indicating reliable set relationships between different conditional combinations and innovation capability.

**Table 8:** Antecedent configuration analysis.

Variable	High			Low	
	1	2	3	1	2
Tool dependence	ⓧ		ⓧ	●	●
Cognitive dependence	ⓧ			●	●
Cognitive inertia	ⓧ	ⓧ	ⓧ	▲	▲
Academic utilitarian atmosphere		ⓧ			●
Employment pressure	ⓧ		ⓧ	▲	▲
Raw coverage	0.687	0.654	0.628	0.212	0.168
Unique coverage	0.062	0.045	0.031	0.076	0.042
Consistency	0.923	0.886	0.861	0.915	0.873
Overall coverage	0.243			0.235	
Overall consistency	0.897			0.889	

● Indicates a necessary condition; ▲ Indicates a core necessary condition; ⓧ Indicates a non-necessary condition; blank cells indicate conditions that may or may not exist.

In the high innovation capability configurations, low cognitive inertia serves as a common core condition across the three paths, demonstrating that maintaining active processing and in-depth thinking is an important psychological foundation for the formation of innovation capability. Configuration 1 exhibits the highest coverage and consistency, making it the most representative path to high innovation. This path presents a synergistic structure of low tool dependence, low cognitive dependence, low cognitive inertia, and moderate employment pressure, revealing a clear behavioral mechanism: when students rely primarily on internal cognitive resources rather than external technology to complete tasks, they are more likely to engage in high-order cognitive activities such as in-depth processing and problem restructuring; meanwhile, moderate employment pressure may provide positive external incentives, prompting individuals to focus on challenging learning tasks and thereby enhancing creative efforts. These conditions collectively point to the core psychological process of active cognitive investment, making this configuration the optimal path to innovation capability.

In the low innovation capability configurations, both configurations exhibit the superimposed characteristics of high tool dependence, high cognitive dependence, high cognitive inertia, and high employment pressure, accompanied by the reinforcing effect of a strong academic utilitarian atmosphere. This combination reflects a typical psychological chain: in high-pressure situations, students are more inclined to rely on AI tools for ready-made answers to reduce time and cognitive consumption; continuous tool dependence further weakens the demand for in-depth processing and catalyzes cognitive inertia; the utilitarian atmosphere strengthens the external evaluation logic of quickly completing tasks, making students more likely to adopt labor-saving and superficial learning strategies, reducing their willingness and behavioral motivation for innovative exploration. The combined effect of the above conditions compresses students’ cognitive resource allocation and thinking space, thereby forming a typical psychological-situational path to low innovation capability.

High innovation paths rely more on active cognitive investment and moderate pressure, while low innovation paths reflect resource-conserving learning strategies jointly driven by technological dependence, pressure intensification, and a utilitarian atmosphere. This result reveals how cognitive traits, situational factors, and technology use form differentiated innovative behavioral mechanisms under different combination conditions, further indicating that innovation capability has multi-path generation characteristics rather than a single causal structure.

## 5 Discussion

Technology Alienation Theory, and Situational Strength Theory, this study systematically explored the mechanism through which AI dependence, cognitive inertia, employment pressure, and academic utilitarian atmosphere affect college students’ innovation capability using PLS-SEM, and further analyzed the path combinations to improve innovation capability by integrating fsQCA.

AI dependence significantly affects college students’ cognitive inertia, and cognitive inertia plays a key mediating role between AI dependence and innovation capability. Among them, the effect of cognitive dependence is stronger than that of tool dependence, indicating that when students gradually transfer cognitive load to AI in the process of thinking and judgment, their investment in in-depth processing and creative thinking decreases significantly. This finding is consistent with existing studies, which point out that the continuous involvement of AI may lead to the outsourcing of students’ thinking, weakening their independent reasoning and critical analysis capabilities (Aboodi, 2025). Different from the conclusion of a few scholars that AI can alleviate cognitive burden and improve learning efficiency in the short term (Feng, 2025), this study, based on large-sample empirical data, reveals the cognitive inertia effect caused by long-term AI dependence, which is more in line with the psychological change law in real learning environments. Theoretically, this result confirms the coupling logic of Technology Alienation Theory and Conservation of Resources Theory: when AI gradually transforms from an auxiliary tool to a cognitive substitute, students’ thinking subjectivity is weakened (Aymen and Zakarya, 2024); and resource threats and cognitive conservation tendencies make individuals inclined to choose low-investment paths (Boon-Falleur et al., 2025). Practically, this study suggests that educational managers should emphasize the thinking framework of human-machine collaboration in AI-empowered teaching, rather than passive dependence on tool substitution. Colleges and universities should design courses oriented toward problem-solving and reflective learning, encourage students to maintain critical scrutiny and self-correction when using AI, prevent inertia from solidifying into learning habits, and thus truly realize the positive empowerment of AI technology on cognitive development.

This study further verified the key moderating roles of employment pressure and academic utilitarian atmosphere in the path of AI dependence – cognitive inertia – innovation capability. The results show that high employment pressure significantly amplifies students’ tendency to conserve resources, prompting them to rely more on AI to reduce learning costs, thereby exacerbating cognitive inertia and weakening innovation investment; while the academic utilitarian atmosphere, by strengthening short-term goals and performance orientation, makes students pay more attention to outcome output and ignore in-depth exploration and reflective learning. This is consistent with existing conclusions that competitive and utilitarian educational environments weaken students’ intrinsic motivation and creative thinking (Chang et al., 2025). Based on Situational Strength Theory, strong situations with clear rewards and punishments and rigid evaluations tend to amplify the constraint effect of the external environment on individual behavior, leading students to be more likely to choose low-investment minimal effort paths under high pressure and utilitarian orientation (Alam et al., 2025). Theoretically, this study expands the contextual boundary of AI dependence research and reveals the interactive logic between psychological factors and the external environment in the formation of innovation capability; practically, the results suggest that colleges and universities should weaken excessive competition and utilitarian evaluation mechanisms through institutional reforms, and build an educational atmosphere centered on exploratory learning, interdisciplinary cooperation, and continuous innovation. At the same time, a psychological support and pressure management system should be established to help students maintain cognitive balance between employment anxiety and AI dependence, and prevent external pressure from evolving into thinking inertia and innovation degradation.

On the basis of the PLS-SEM model, the fsQCA results further revealed the multi-path generation mechanism of college students’ innovation capability. The study identified a variety of effective conditional combinations leading to high innovation capability, indicating that innovation performance is not determined by a single factor but is jointly shaped by the synergistic effect of AI dependence, cognitive inertia, and the external environment. Among them, the combination of low tool dependence, low cognitive dependence, low cognitive inertia, and moderate employment pressure constitutes the optimal conditional configuration for promoting innovation, suggesting that moderate pressure and limited technological dependence can jointly stimulate students’ cognitive investment, thereby enhancing innovation motivation (Braun, 2025). On the contrary, the combination of high AI dependence, high cognitive inertia, high employment pressure, and a strong utilitarian atmosphere is significantly negatively correlated with innovation capability, indicating that when external pressure is superimposed with the abuse of technology, the space for students’ creative thinking is inhibited (Kuzmanov, 2025). This confirms the crucial influence of the synergy between intrinsic cognitive autonomy and external context adaptability on innovation capability. It not only points out the core role of reducing various dependencies and breaking cognitive inertia in stimulating innovation capability, but also emphasizes that the external context should avoid excessive stress and excessive utilitarianism. By balancing incentives and inclusiveness, long-term values and short-term goals, individual innovation capabilities can be enhanced. Theoretically, this study revealed the multi-path logic of innovation capability formation through fsQCA, verified the interactive applicability of Social Cognitive Theory, Conservation of Resources Theory, and Technology Alienation Theory, and expanded their application boundary in the research of complex educational behaviors. Practically, the results provide path references for educators in differentiated training: for groups with high dependence and high pressure, inertial paths should be reduced through teaching design and task restructuring; for students with AI application capabilities and in moderate pressure situations, goal-oriented learning and AI-assisted training can be used to stimulate innovation potential.

## 6 Limitations and implications

### 6.1 Theoretical implications

From a multi-theoretical perspective, this study provides important theoretical implications for understanding the development mechanism of AI dependence and innovation capability, not only verifying the explanatory power of classic theories but also expanding their application boundary and connotative depth in the field of educational technology. The research findings confirm that AI dependence has a significant positive impact on cognitive inertia, and cognitive inertia plays a key mediating role between AI dependence and innovation capability, further enriching the cognitive formation mechanism under the interaction between individuals and the technological environment. As a new type of environmental variable, AI technology’s impact on individual cognition is not one-way empowerment, but may also reduce college students’ willingness to take initiative in thinking and in-depth processing, supplementing the internal logic of dynamic interaction between individuals, behaviors, and the environment in Social Cognitive Theory. At the same time, this study confirms the applicability of Technology Alienation Theory at the cognitive level, breaking through the previous single understanding that technology alienation is a direct negative impact, and reveals the mechanism through which AI dependence weakens innovation capability by inducing cognitive inertia, providing a new explanatory path for the micro-application of Technology Alienation Theory in educational cognitive research. In addition, the moderating effects of employment pressure and academic utilitarian atmosphere verify the core viewpoint of Situational Strength Theory, that is, the higher the external situational strength, the more likely individual behavior is to be constrained by the situation; at the same time, this study expands the theoretical boundary of situational variables in the relationship between AI technology and cognition, extending the impact of situational strength to the process of technology use and cognitive development in educational scenarios, providing a theoretical basis for understanding the differentiated impact of technology on cognitive effects under different external environments, and also providing a theoretical reference for the subsequent formulation of intervention and teaching strategies for different educational situations.

### 6.2 Managerial implications

From the perspective of educational management and teaching practice, this study has important reference value for colleges and universities to improve students’ innovation capability in the AI era. Firstly, educators should recognize the dual-edged nature of AI tools and regard them as cognitive boosters rather than thinking substitutes. Teachers should integrate AI literacy education and reflective use training into teaching to help students establish the ability of critical evaluation and in-depth processing of AI results. Secondly, schools need to optimize the academic evaluation system, downplay short-term utilitarian assessments, promote the transformation of teaching from result-oriented to process-oriented, and stimulate students’ spirit of exploration and interest in innovation. Attention should also be paid to the impact of employment pressure on students’ psychology and learning behaviors, and a multi-level support system should be established to alleviate anxiety and strengthen creative investment through innovative experimental courses, interdisciplinary projects, and career counseling. Finally, families and society should form an inclusive and supportive innovation ecosystem, encouraging students to maintain independent thinking and continuous exploration in the AI-assisted learning environment, and preventing the long-term erosion of innovation potential by excessive educational utilitarianism.

### 6.3 Research limitations and future directions

Despite the valuable progress made in both theoretical and empirical aspects, this study still has several limitations. First, it adopts cross-sectional data, which limits the accuracy of causal inference and prevents full capture of the dynamic evolutionary process among AI dependence, cognitive inertia, and innovation capability. Future research can further verify these causal paths through longitudinal designs, experimental interventions, or mixed methods. Second, this study mainly relies on self-reported questionnaires, which may be affected by social desirability bias or memory bias. To improve the objectivity and robustness of measurements, subsequent studies can integrate behavioral tracking data, AI usage logs, or third-party evaluation indicators. Third, the sample of this study uses stratified convenience sampling. Although it covers various types of universities and disciplines, the external representativeness remains limited. Future research can adopt multi-center random sampling or international comparative studies to enhance external validity. Fourth, this study takes Chinese college students as samples, and their educational system and cultural background may affect the generalizability of the results. In the future, comparative studies can be conducted under different cultures and educational systems to explore the cross-cultural differences between AI dependence, cognitive inertia, and innovation capability. Finally, at the methodological level, this study has not fully controlled for potential confounding variables, such as individual personality, disciplinary characteristics, or learning motivation, which may influence the relationship between AI dependence and innovation capability. Future research can introduce control variables in the design or use latent variable adjustment in structural equation modeling to improve the robustness of the conclusions.

## 7 Conclusion

Based on a sample of 1,032 college students, this study systematically explored the impact mechanism of AI dependence, cognitive inertia, employment pressure, and academic utilitarian atmosphere on college students’ innovation capability using PLS-SEM and fsQCA, and further analyzed the optimal combination path to improve innovation capability. The results show that AI dependence has a significant positive impact on cognitive inertia, with the effect of cognitive dependence being stronger than that of tool dependence; cognitive inertia plays a key mediating role between AI dependence and innovation capability, indicating that excessive reliance on external intelligence weakens students’ in-depth thinking and creative exploration, thereby reducing innovation performance.

The external situation plays a significant moderating role in this mechanism. Employment pressure strengthens the positive impact of AI dependence on cognitive inertia, while the academic utilitarian atmosphere amplifies the inhibitory effect of cognitive inertia on innovation capability. The fsQCA results further reveal that the formation of innovation capability is the result of the synergistic effect of multiple factors, rather than the linear effect of a single variable. Among them, the combination of low tool dependence, low cognitive dependence, low cognitive inertia, and moderate employment pressure constitutes the optimal path, which can maintain moderate psychological pressure while reducing the excessive use of technology, and promote the release of innovation potential.

Theoretically, this study integrates Social Cognitive Theory, Conservation of Resources Theory, Technology Alienation Theory, and Situational Strength Theory to construct a framework linking AI dependence, cognitive inertia, and innovation capability. It also highlights the boundary effects of environmental factors, thereby extending theoretical perspectives in educational technology and innovation psychology. The findings underscore that AI’s role in education is not unidirectional; its impact depends on the dynamic interplay between individual cognitive states and the external environment. Practically, educators should guide students to use AI tools purposefully while preserving independent thinking and critical analysis. Universities should optimize academic evaluation systems, alleviate employment pressure, and foster supportive learning environments to enhance students’ creativity and sustained innovation. Future research could further test the model’s robustness and generalizability using longitudinal and cross-cultural samples, offering theoretical and practical insights for cultivating innovative talent and reforming higher education.

## Data Availability

The original contributions presented in the study are included in the article/Supplementary material, further inquiries can be directed to the corresponding author.
