# Supplementary material for: The impact mechanism of artificial intelligence dependence on college students’ innovation capability: an empirical study from China
Source: Front Psychol. 2025 Dec 12;16:1732837. doi: 10.3389/fpsyg.2025.1732837 (PMC12742215; doi:10.3389/fpsyg.2025.1732837)
Supplement: Supplementary file 1 [file Supplementary_file_1.docx]

Supplementary Material

The Impact Mechanism of Artificial Intelligence Dependence on College Students' Innovation Capability: An Empirical Study from China

**Zhixin Yang^1^, Hao Deng^1*^, Nan Jiang^1^**

*** Correspondence:** Hao Deng: yangzhixin97@126.com

**Appendix 1**

**1 Statistical Methods**

This study employed partial least squares structural equation modeling (PLS-SEM), a symmetric or linear approach, and fuzzy-set qualitative comparative analysis (fsQCA), an asymmetric or non-linear approach, to analyze the data. These two methods have different focuses and are based on different principles. PLS-SEM is a variance-based technique aimed at developing theories by predicting and maximizing the explained variance in dependent variables (Hair et al., 2019). Compared to covariance-based SEM, PLS-SEM offers flexibility in modeling complex structures and accommodates small samples and non-normal data distributions (Hair et al., 2019). Furthermore, this study utilized fsQCA as a complementary method to enhance the robustness of the findings. The fsQCA analysis follows the configuration theory paradigm, leveraging Boolean logic to identify multiple solutions leading to a specific outcome (Chuah et al., 2021). FsQCA handles non-linearity and complex complementarities between independent and dependent variables, rather than simply identifying correlations between them (e.g., regression analysis and SEM) (Gil-Cordero et al., 2024). Additionally, fsQCA helps differentiate between necessary and sufficient causal conditions for an outcome. We performed the fsQCA algorithm using the QCA package (version 3.23; Dusa, 2024) and conducted the PLS-SEM analysis using Smart PLS 4.0 (Hair et al., 2019).

**1.1 PLS-SEM approach**

PLS-SEM analysis consists of two stages: first, the assessment of the measurement model, and second, hypothesis testing using the structural model. All HBM constructs were specified as reflective constructs, as their measurement items are assumed to be manifestations of the underlying latent constructs. The measurement model was assessed in terms of internal consistency, reliability, and validity (Hair et al., 2019). We evaluated the internal consistency reliability of the constructs using two criteria: Cronbach's alpha and composite reliability. The recommended threshold for these criteria is greater than 0.70 (Hair et al., 2019). Model validity was determined through convergent validity and discriminant validity. We assessed convergent validity using the Average Variance Extracted (AVE). AVE measures the degree of variance in the observed variables associated with their respective constructs, with a threshold greater than 0.50 (Hair et al., 2019). We used the Fornell-Larcker criterion to assess discriminant validity (Abbasi et al., 2022). Discriminant validity is established when the square root of the AVE for each construct is greater than its correlations with other constructs. Finally, multicollinearity was assessed using the Variance Inflation Factor (VIF), with a threshold of < 5.

To assess the significance of the hypothesized relationships in the structural model, a non-parametric, bias-corrected bootstrap procedure with 5000 subsamples and 95% confidence intervals was employed (Hair et al., 2019). Unlike covariance-based structural equation modeling, which relies on goodness-of-fit indices, the partial least squares method evaluates the structural model by examining path coefficients, t-values, the coefficient of determination (R²), the effect size of path coefficients (f²), and predictive relevance (Q²). R² measures the proportion of variance in the dependent variable (PB) explained by the model, with values of 0.75, 0.50, and 0.25 indicating substantial, moderate, and weak explanatory power, respectively (Hair et al., 2019). f² assesses the effect size of each independent variable (predictor) on the dependent variable, explaining the unique variance attributed to the predictor rather than shared variances (Welkowitz et al., 2011).

**1.2 FsQCA approach**

We further employed fsQCA to examine the synergistic effects of predictors on protective behavior. This method involves three main steps. Calibration is the first crucial step, which transforms the raw scores of dependent and independent variables into values reflecting set membership, ranging from 0 (full non-membership) to 1 (full membership), with intermediate scores indicating partial membership (Thygeson et al., 2011). Calibration can be performed in two ways: indirect calibration, which involves qualitatively assessing cases and rescaling the underlying measurements, and direct calibration, which involves identifying three distinct membership anchors in a set (Diwanji, 2023).

The second step is the analysis of necessary conditions. A condition is considered necessary if its presence always leads to the outcome; however, its presence alone does not guarantee the outcome (Abbasi et al., 2022). A configuration is deemed necessary when the consistency score exceeds 0.90 (Janse van Rensburg et al., 2021). The final step is the assessment of the truth table or sufficiency analysis, aimed at identifying the various combinations of causal conditions that are sufficient for achieving a high or low level of PB. The fsQCA algorithm generates a truth table with 2^k rows, where k denotes the number of causal conditions, and each row corresponds to a unique combination of these conditions (Huarng & Roig-Tierno, 2016). To determine sufficient configurations, we applied thresholds of consistency > 0.90, coverage > 0.20 (Janse van Rensburg et al., 2021), and a PRI score > 0.7 (Huarng & Roig-Tierno, 2016). Consistency measures the degree to which cases correspond to the set-theoretic relationships expressed in a solution, analogous to the path coefficient (β). Coverage measures the extent to which a causal solution accounts for the outcome, similar to R², and the Proportional Reduction in Inconsistency (PRI) assesses the degree to which a condition consistently contributes to the outcome.

**Appendix 2**

Descriptive Analysis and Normality Test of Each Item

| Item | Mean | Standard Deviation | Skewness | Kurtosis |
| --- | --- | --- | --- | --- |
| ATD1 | 4.05 | 1.033 | -1.119 | 0.819 |
| ATD2 | 4.00 | 0.984 | -1.100 | 1.000 |
| ATD3 | 4.05 | 0.996 | -1.132 | 0.992 |
| ATD4 | 3.95 | 1.065 | -1.04 | 0.526 |
| ATD5 | 4.07 | 1.036 | -1.197 | 1.03 |
| ATD6 | 4.04 | 0.981 | -1.146 | 1.129 |
| ATD7 | 4.06 | 0.997 | -1.150 | 1.023 |
| ATD8 | 3.94 | 1.105 | -1.047 | 0.425 |
| ATD9 | 4.16 | 0.958 | -1.258 | 1.472 |
| ATD10 | 4.06 | 0.925 | -1.094 | 1.222 |
| ATD11 | 4.09 | 0.962 | -1.146 | 1.192 |
| ATD12 | 3.99 | 1.064 | -1.135 | 0.767 |
| ACD1 | 4.00 | 1.102 | -1.195 | 0.789 |
| ACD2 | 3.95 | 1.133 | -1.147 | 0.651 |
| ACD3 | 3.96 | 1.127 | -1.189 | 0.751 |
| ACD4 | 3.96 | 1.112 | -1.116 | 0.638 |
| ACD5 | 4.00 | 1.107 | -1.227 | 0.915 |
| ACD6 | 3.97 | 1.113 | -1.115 | 0.588 |
| ACD7 | 3.99 | 1.100 | -1.163 | 0.787 |
| ACD8 | 3.91 | 1.130 | -1.037 | 0.365 |
| ACD9 | 3.97 | 1.097 | -1.130 | 0.700 |
| CI1 | 3.97 | 1.136 | -1.242 | 0.851 |
| CI2 | 3.95 | 1.133 | -1.224 | 0.843 |
| CI3 | 3.92 | 1.143 | -1.154 | 0.639 |
| CI4 | 3.99 | 1.154 | -1.296 | 0.951 |
| CI5 | 3.95 | 1.141 | -1.227 | 0.802 |
| CI6 | 4.00 | 1.124 | -1.286 | 1.010 |
| CI7 | 3.96 | 1.120 | -1.245 | 0.935 |
| CI8 | 3.97 | 1.126 | -1.235 | 0.883 |
| CI9 | 4.01 | 1.150 | -1.306 | 0.980 |
| SUA1 | 3.88 | 1.143 | -1.037 | 0.338 |
| SUA2 | 3.88 | 1.079 | -0.989 | 0.381 |
| SUA3 | 3.78 | 1.126 | -0.758 | -0.196 |
| SUA4 | 3.80 | 1.102 | -0.874 | 0.132 |
| SUA5 | 3.84 | 1.120 | -0.965 | 0.231 |
| SUA6 | 3.88 | 1.137 | -0.989 | 0.239 |
| SUA7 | 3.87 | 1.132 | -0.869 | -0.054 |
| SUA8 | 3.92 | 1.136 | -0.967 | 0.133 |
| SUA9 | 3.89 | 1.106 | -0.925 | 0.123 |
| EP1 | 3.99 | 1.065 | -1.035 | 0.356 |
| EP2 | 4.04 | 1.015 | -1.054 | 0.538 |
| EP3 | 4.10 | 1.009 | -1.144 | 0.626 |
| EP4 | 4.00 | 1.020 | -0.978 | 0.314 |
| EP5 | 4.09 | 0.970 | -1.063 | 0.585 |
| EP6 | 4.07 | 1.010 | -1.087 | 0.631 |
| EP7 | 4.03 | 1.017 | -0.969 | 0.194 |
| EP8 | 4.05 | 1.010 | -1.029 | 0.441 |
| EP9 | 4.06 | 0.983 | -1.093 | 0.669 |
| CSIA1 | 3.85 | 1.099 | -0.805 | -0.154 |
| CSIA2 | 3.85 | 1.081 | -0.854 | 0.093 |
| CSIA3 | 3.83 | 1.065 | -0.802 | -0.027 |
| CSIA4 | 3.81 | 1.106 | -0.798 | -0.094 |
| CSIA5 | 3.85 | 1.100 | -0.835 | -0.116 |
| CSIA6 | 3.84 | 1.074 | -0.824 | 0.010 |
| CSIA7 | 3.88 | 1.086 | -0.901 | 0.162 |
| CSIA8 | 3.82 | 1.075 | -0.827 | 0.015 |
| CSIA9 | 3.82 | 1.073 | -0.809 | 0.006 |
| CSIA10 | 3.83 | 1.097 | -0.836 | -0.020 |
| CSIA11 | 3.82 | 1.100 | -0.798 | -0.165 |
| CSIA12 | 3.68 | 1.178 | -0.660 | -0.487 |
| CSIA13 | 3.74 | 1.191 | -0.770 | -0.378 |
| CSIA14 | 3.75 | 1.166 | -0.730 | -0.400 |
| CSIA15 | 3.74 | 1.179 | -0.778 | -0.287 |
| CSIA16 | 3.77 | 1.194 | -0.780 | -0.370 |
| CSIA17 | 3.66 | 1.169 | -0.618 | -0.535 |
| CSIA18 | 3.68 | 1.194 | -0.676 | -0.489 |
| CSIA19 | 3.66 | 1.184 | -0.656 | -0.539 |
| CSIA20 | 3.67 | 1.129 | -0.633 | -0.486 |
| CSIA21 | 3.69 | 1.139 | -0.721 | -0.310 |
| CSIA22 | 3.70 | 1.175 | -0.727 | -0.350 |
| CSIA23 | 3.72 | 1.213 | -0.735 | -0.427 |
| CSIA24 | 3.71 | 1.141 | -0.650 | -0.465 |
| CSIA25 | 3.73 | 1.131 | -0.682 | -0.367 |

**Appendix 3**

Factor Loadings and VIF

| Item | Innovation Capability | Academic Utilitarian Atmosphere | Employment Pressure | Tool Dependence | Cognitive Dependence | Cognitive Inertia | VIF |
| --- | --- | --- | --- | --- | --- | --- | --- |
| ACD1 |  |  |  |  | 0.757 |  | 1.934 |
| ACD2 |  |  |  |  | 0.763 |  | 1.927 |
| ACD3 |  |  |  |  | 0.753 |  | 1.898 |
| ACD4 |  |  |  |  | 0.755 |  | 1.891 |
| ACD5 |  |  |  |  | 0.776 |  | 2.309 |
| ACD6 |  |  |  |  | 0.751 |  | 1.931 |
| ACD7 |  |  |  |  | 0.770 |  | 2.090 |
| ACD8 |  |  |  |  | 0.751 |  | 1.919 |
| ACD9 |  |  |  |  | 0.819 |  | 2.915 |
| ATD1 |  |  |  | 0.687 |  |  | 1.985 |
| ATD10 |  |  |  | 0.640 |  |  | 1.654 |
| ATD11 |  |  |  | 0.651 |  |  | 1.615 |
| ATD12 |  |  |  | 0.621 |  |  | 1.608 |
| ATD2 |  |  |  | 0.687 |  |  | 1.818 |
| ATD3 |  |  |  | 0.655 |  |  | 1.699 |
| ATD4 |  |  |  | 0.652 |  |  | 1.974 |
| ATD5 |  |  |  | 0.647 |  |  | 2.252 |
| ATD6 |  |  |  | 0.644 |  |  | 1.835 |
| ATD7 |  |  |  | 0.625 |  |  | 1.74 |
| ATD8 |  |  |  | 0.601 |  |  | 1.914 |
| ATD9 |  |  |  | 0.675 |  |  | 1.905 |
| CI1 |  |  |  |  |  | 0.830 | 2.553 |
| CI2 |  |  |  |  |  | 0.823 | 2.450 |
| CI3 |  |  |  |  |  | 0.817 | 2.472 |
| CI4 |  |  |  |  |  | 0.825 | 2.526 |
| CI5 |  |  |  |  |  | 0.822 | 2.495 |
| CI6 |  |  |  |  |  | 0.723 | 2.381 |
| CI7 |  |  |  |  |  | 0.733 | 2.350 |
| CI8 |  |  |  |  |  | 0.739 | 2.412 |
| CI9 |  |  |  |  |  | 0.697 | 2.219 |
| CSIA1 | 0.741 |  |  |  |  |  | 2.486 |
| CSIA10 | 0.727 |  |  |  |  |  | 2.375 |
| CSIA11 | 0.744 |  |  |  |  |  | 2.689 |
| CSIA12 | 0.712 |  |  |  |  |  | 2.464 |
| CSIA13 | 0.711 |  |  |  |  |  | 2.460 |
| CSIA14 | 0.680 |  |  |  |  |  | 2.980 |
| CSIA15 | 0.662 |  |  |  |  |  | 2.597 |
| CSIA16 | 0.686 |  |  |  |  |  | 2.738 |
| CSIA17 | 0.717 |  |  |  |  |  | 2.893 |
| CSIA18 | 0.718 |  |  |  |  |  | 2.509 |
| CSIA19 | 0.701 |  |  |  |  |  | 2.353 |
| CSIA2 | 0.730 |  |  |  |  |  | 2.334 |
| CSIA20 | 0.682 |  |  |  |  |  | 2.178 |
| CSIA21 | 0.615 |  |  |  |  |  | 2.163 |
| CSIA22 | 0.605 |  |  |  |  |  | 2.066 |
| CSIA23 | 0.615 |  |  |  |  |  | 2.449 |
| CSIA24 | 0.732 |  |  |  |  |  | 2.565 |
| CSIA25 | 0.724 |  |  |  |  |  | 2.297 |
| CSIA3 | 0.747 |  |  |  |  |  | 2.725 |
| CSIA4 | 0.750 |  |  |  |  |  | 2.650 |
| CSIA5 | 0.743 |  |  |  |  |  | 2.336 |
| CSIA6 | 0.746 |  |  |  |  |  | 2.466 |
| CSIA7 | 0.766 |  |  |  |  |  | 2.701 |
| CSIA8 | 0.761 |  |  |  |  |  | 2.668 |
| CSIA9 | 0.708 |  |  |  |  |  | 2.593 |
| EP1 |  |  | 0.707 |  |  |  | 2.016 |
| EP2 |  |  | 0.749 |  |  |  | 2.368 |
| EP3 |  |  | 0.672 |  |  |  | 2.110 |
| EP4 |  |  | 0.678 |  |  |  | 1.848 |
| EP5 |  |  | 0.722 |  |  |  | 2.014 |
| EP6 |  |  | 0.720 |  |  |  | 2.035 |
| EP7 |  |  | 0.708 |  |  |  | 2.498 |
| EP8 |  |  | 0.761 |  |  |  | 3.046 |
| EP9 |  |  | 0.721 |  |  |  | 2.822 |
| SUA1 |  | 0.795 |  |  |  |  | 2.134 |
| SUA2 |  | 0.756 |  |  |  |  | 1.946 |
| SUA3 |  | 0.716 |  |  |  |  | 1.720 |
| SUA4 |  | 0.775 |  |  |  |  | 1.993 |
| SUA5 |  | 0.768 |  |  |  |  | 1.975 |
| SUA6 |  | 0.781 |  |  |  |  | 2.056 |
| SUA7 |  | 0.759 |  |  |  |  | 1.909 |
| SUA8 |  | 0.770 |  |  |  |  | 1.935 |
| SUA9 |  | 0.733 |  |  |  |  | 1.779 |

Abbasi, G. A., Sandran, T., Ganesan, Y., & Iranmanesh, M. (2022). Go cashless! Determinants of continuance intention to use E-wallet apps: A hybrid approach using PLS-SEM and fsQCA. *Technology in Society*, *68*, 101937.

Chuah, S. H.-W., Tseng, M.-L., Wu, K.-J., & Cheng, C.-F. (2021). Factors influencing the adoption of sharing economy in B2B context in China: Findings from PLS-SEM and fsQCA. *Resources, Conservation and Recycling*, *175*, 105892.

Diwanji, V. S. (2023). Fuzzy‐set qualitative comparative analysis in consumer research: A systematic literature review. *International Journal of Consumer Studies*, *47*(6), 2767-2789.

Gil-Cordero, E., Ledesma-Chaves, P., Arteaga Sánchez, R., & Mariano, A. M. (2024). Crypto-wallets revolution! Key factors driving behavioral intention to adopt the Coinbase Wallet using mixed PLS-SEM/fsQCA methodology in the Spanish environment. *International Journal of Bank Marketing*, *42*(3), 536-570.

Hair, J. F., Risher, J. J., Sarstedt, M., & Ringle, C. M. (2019). When to use and how to report the results of PLS-SEM. *European Business Review*, *31*(1), 2-24.

Huarng, K.-H., & Roig-Tierno, N. (2016). Qualitative comparative analysis, crisp and fuzzy sets in knowledge and innovation. *Journal of Business Research*, *69*(11), 5181-5186.

Janse van Rensburg, A., Kathree, T., Breuer, E., Selohilwe, O., Mntambo, N., Petrus, R.,…Petersen, I. (2021). Fuzzy-set qualitative comparative analysis of implementation outcomes in an integrated mental healthcare trial in South Africa. *Global health action*, *14*(1), 1940761.

Thygeson, N. M., Solberg, L. I., Asche, S. E., Fontaine, P., Pawlson, L. G., & Scholle, S. H. (2011). Using fuzzy set qualitative comparative analysis (fs/QCA) to explore the relationship between medical “homeness” and quality. *Health services research*, *47*(1 Pt 1), 22.

Welkowitz, J., Cohen, B. H., & Lea, R. B. (2011). *Introductory statistics for the behavioral sciences*. John Wiley & Sons.
